# Oral energy supplementation improves nutritional status in hemodialysis patients with protein–energy wasting: A pilot study

**DOI:** 10.3389/fphar.2022.839803

**Published:** 2022-10-21

**Authors:** Aiya Qin, Jiaxing Tan, Wen Hu, Yuan Liu, Lin Chen, Yi Tang, Wei Qin

**Affiliations:** ^1^ Division of Nephrology, Department of Medicine, West China Hospital, Sichuan University, Chengdu, Sichuan, China; ^2^ West China School of Medicine, Sichuan University, Chengdu, Sichuan, China; ^3^ Department of Clinical Nutrition, West China Hospital, Sichuan University, Chengdu, China; ^4^ Hemodialysis Center, Department of Nephrology, West China Hospital/West China School of Nursing, Sichuan University, Chengdu, China

**Keywords:** protein–energy wasting, hemodialysis, oral energy supplements, HD, PEW

## Abstract

**Background:** Protein–energy wasting (PEW) is highly prevalent in hemodialysis (HD) patients, which is associated with poor quality of life, complications, and an increased risk of mortality. A prospective study in HD patients with 2 months of oral energy supplements (OESs) was performed.

**Methods:** A total of 37 HD patients with PEW were finally enrolled in this prospective study and were randomized into the OES group (*n* = 19), which received oral energy supplementation (300 kcal) and dietary recommendations, while patients in the non-OES group (*n* = 18) received only dietary recommendations. The study duration was 2 months. The nutritional status of the patients was evaluated by laboratory indexes, body composition parameters, and the modified quantitative subjective global assessment (MQSGA) and malnutrition-inflammation score (MIS). Quality of life was evaluated by the Short Form Health Survey Questionnaire (SF-36).

**Results:** After 2 months of therapy, a significant increase in serum albumin [39.6 (37.6–45.8) vs. 43.4 (39.1–46.7) g/L; *p =* 0.018], hemoglobin (101.0 ± 13.6 g/L vs. 111.8 ± 11.7 g/L; *p =* 0.042), and dietary energy intake (29.17 ± 3.22 kcal/kg/day vs. 33.60 ± 2.72 kcal/kg/day, *p* < 0.001) was observed in the comparisons of baseline in the OES group. Moreover, the OES group demonstrated significant amelioration in MQSGA [9 (8–13) vs. 8 (7–12), *p* < 0.001] and MIS [5 (3–10) vs. 3 (2–8), *p* < 0.001], physical functioning (*p* < 0.001), and mental health (*p =* 0.046) subsections of SF-36 compared with the baseline. No electrolyte disorders or dyslipidemia were observed in the OES group.

**Conclusion:** OES in HD patients with PEW can significantly ameliorate energy supply, nutritional status, anemia, and quality of life.

## Introduction

Protein energy wasting (PEW) is a state of metabolic and nutritional derangements in chronic kidney disease (CKD) and is one of the most common comorbidities, with a prevalence of 18%–75% in patients undergoing maintenance dialysis therapy (Małgorzewicz et al., 2019). Emerging as a progressive depletion of individual protein and energy stores, this condition is associated with poor quality of life, complications, and an increased risk of mortality (Qureshi et al., 2002). The Kidney Disease Outcomes Quality Initiative nutritional (KDOQI) guidelines have recommended hemodialysis (HD) patients with 30–35 kcal/kg/day of energy intake (Ikizler et al., 2020). However, many patients fail to comply with these recommendations due to dietary restrictions, anorexia, and socioeconomic limitations (Wu et al., 2013; Sabatino et al., 2018; Bolasco, 2020). Additional intervention is needed to aid patients in achieving therapeutic nutritional energy goals, in which oral energy supplements (OESs) may offer benefits over protein- or carbohydrate-dense supplements for patients undergoing dialysis because of the adverse metabolic consequences of the latter (Yang et al., 2021). However, previous studies had considered oral nutritional supplements, which contain proteins and electrolytes. Few studies have considered the influence of OES on HD patients (Lacson et al., 2007; Moretti et al., 2009; Sezer et al., 2014; Weiner et al., 2014; Benner et al., 2018; Leonberg-Yoo et al., 2019; Limwannata et al., 2021). Thus, this study aimed to evaluate the effects of OES on nutritional status in HD patients.

## Materials and methods

### Study population

Patients were recruited from the Hemodialysis Center of West China Hospital, Sichuan University, from July 2019 to September 2019. Inclusion criteria for this study were as follows: 1) age between 18 and 90 years, regular hemodialysis treatment for 4 hours, three times weekly for ≥3 months; 2) absence of infection; 3) diagnosed as PEW according to the criteria introduced by the International Society of Renal Nutrition and Metabolism (Fouque et al., 2008); 4) written informed consent and ability to understand the study protocol. We randomly assigned patients into the OES group and the non-OES group. This study was registered at the Clinical Trial Registration Center of Thailand (https://thaiclinicaltrials.org;TCTR20180313004) and was approved by the Ethical Committee of West China Hospital of Sichuan University (2019–33). Informed consent was obtained from each patient or legal guardians prior to treatment.

### Nutritional intervention

All participants received dietary counseling as recommended by the KDOQI guidelines (1.0–1.2 g/kg/day of protein and 30–35 kcal/kg/day of energy) (Ikizler et al., 2020). Erythropoietin-stimulating agents were prescribed, and the therapeutic dose was not adjusted during the study period. Patients were divided into two groups: the OES group received dietary recommendations plus OES (at a dose of 30 ml twice a day, which supplied 300 kcal of energy) for 2 consecutive months, and the non-OES group only received dietary recommendations for the same duration. Fresubin (Fresenius Kabi, Beijing, China) was applied as the OES in this study. One bottle of OES (120 ml) contains 600 kcal of energy, 4.0 g of carbohydrates, and 53.8 g of lipids, with no content of potassium, protein, and phosphorus and a reduced content of sodium (18 mg). A clinical dietitian instructed both groups and provided dietary counseling during the study.

### Laboratory parameters

Blood samples from patients were collected before hemodialysis to measure serum creatinine (Cr), blood glucose (Glu), hemoglobin (Hb), serum albumin (Alb), blood urea nitrogen (BUN), triglycerides (TGs), cholesterol (TC), high-density lipoprotein (HDL), low-density lipoprotein (LDL), uric acid (UA), parathyroid hormone (PTH), serum calcium (Ca), serum phosphorus (P), and serum kalium (K), using standard laboratory testing procedures.

### Evaluation of nutritional status

Anthropometric measurements were obtained by a trained nurse after the completion of the hemodialysis sessions. Skin-fold (TSF) thickness was measured by using a skin-fold caliper. Mid-arm circumference (MAC) was measured (in centimeter) with a tape measure. The body mass index (BMI) was calculated according to the following equation: BMI = body weight/height^2^. Dietary protein intake (DPI) and dietary energy intake (DEI) values were determined from one 72-h diet recall by the dietitian and analyzed for nutrition composition using the Australian Food Composition Database. The modified quantitative subjective global assessment (MQSGA) and malnutrition inflammation-score (MIS) were chosen as nutrition screening tools by face-to-face interviews as previously reported (Ikizler et al., 2020).

### Health-related quality of life

HRQOL was evaluated by the Short Form Health Survey Questionnaire (SF-36), which included 36 items measuring participant health status in eight dimensions. Each dimension contained questions precoded numerically, transformable to values of 0–100, with a higher score meaning a better quality of life (Li et al., 2018).

### Statistical analysis

Continuous variables were expressed as the mean ± standard deviation (SD). Categorical data were presented as frequencies (percentages). According to the data distribution, Student’s t-test was used to perform pre- and post-intervention comparisons for parametric distributions, and the Mann–Whitney U-test or Wilcoxon tests were used for nonparametric distributions. All of these data were analyzed using the SPSS 23.0 software package (SPSS, Chicago, IL, Unites States), and *p* values were calculated as two-sided. The statistical significance level was set at *p* < 0.05.

## Results

### Baseline characteristics

**FIGURE 1:**
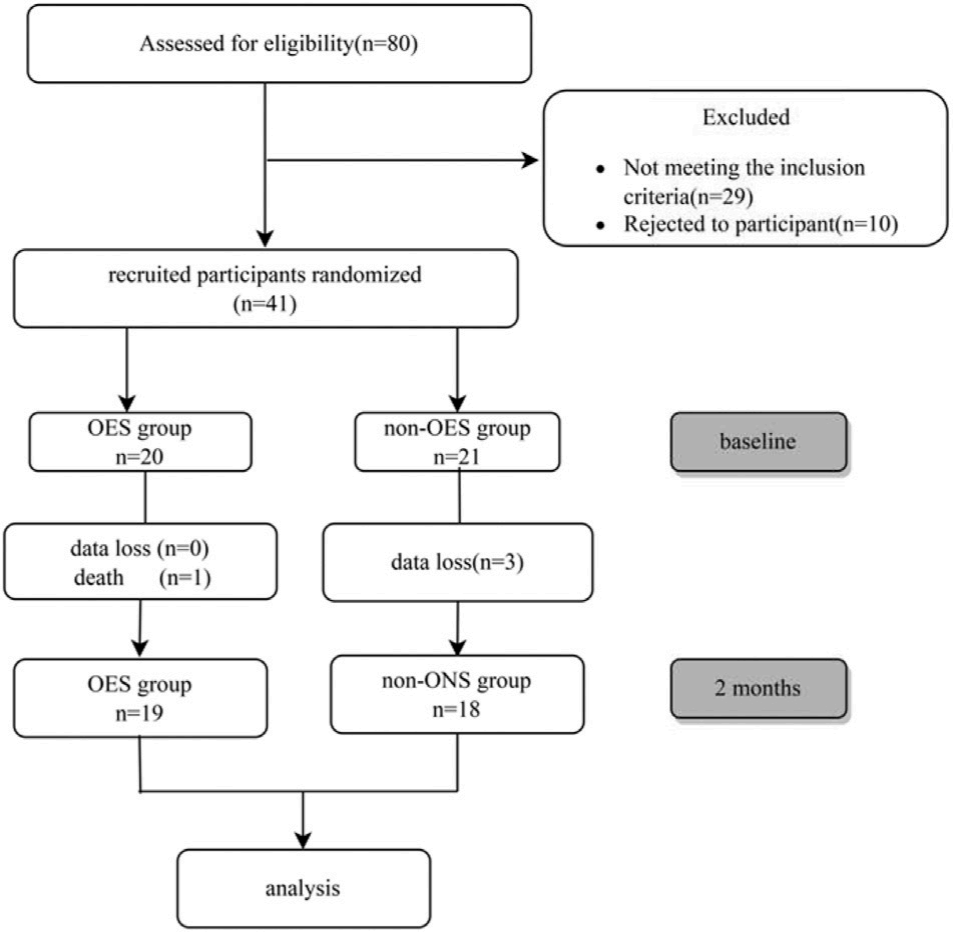
Flow chart of patient enrollment.

According to the inclusion and exclusion criteria, out of 80 individuals screened for inclusion, 51 were eligible, of whom 10 declined to take part, resulting in 41 being included in the study and randomized to the intervention or control groups from July 2019 to September 2019. During the study, one patient in the OES group died of an acute cerebrovascular event, and three patients in the non-OES group dropped out due to data loss. Finally, a total of 37 HD patients (16 females) with a mean age of 63.4 ± 14.8 years were enrolled (Figure 1), in which 19 patients were treated with OES (300 kcal/day) plus dietary recommendations and 18 patients were treated only with dietary recommendations. Both groups continued the treatment for 2 consecutive months. The demographic, biochemical, and clinical characteristics of patients between the two groups are shown in Table 1. No significant differences were observed between the two groups in gender, nutritional status, or laboratory parameters.

**TABLE 1:** Demographic, anthropometric, and nutritional characteristics of the study population.

	All	OES group	Non-OES group	*P*
N	37	19	18	
Age (years)	63.4 ± 14.8	68.2 ± 12.0	58.4 ± 16.1	0.043
Gender, female	16 (43.2)	8 (43.1)	8 (44.4)	0.560
Dialysis time (years)	4.1 ± 3.6	3.1 ± 3.1	5.1 ± 3.8	0.083
BMI (kg/m^2^)	22.7 (20.7–25.0)	23.0 (20.9–26.2)	22.5 (20.5–23.9)	0.605
MIS	5 (3–8.5)	5 (3–10)	5 (2–8)	0.193
MQSGA	9.5 (8–13)	9 (8–13)	10 (8–12)	0.575
Hb (g/L)	104.14 ± 13.83	101.00 ± 13.64	107.44 ± 13.62	0.159
Alb (g/L)	40.7 (37.1–45.6)	39.6 (37.6–45.8)	41.9 (36.2–45.5)	0.855
TC (mmol/L)	3.61 ± 0.84	3.35 ± 0.678	3.87 ± 0.92	0.057
LDL (mmol/L)	1.88 ± 0.67	1.75 ± 0.69	2.02 ± 0.64	0.233
TG (mmol/L)	1.30 (0.78–1.91)	1.14 (0.74–1.80)	1.63 (1.03–2.06)	0.181
HDL (mmol/L)	0.96 (0.80–1.27)	0.97 (0.78–1.33)	0.96 (0.79–1.25)	0.584
Cr (umol/L)	659.1 ± 247.7	679.1 ± 262.6	638.0 ± 236.6	0.621
BUN (mmol/L)	19.0 ± 8.6	20.5 ± 9.0	17.4 ± 8.3	0.292
Glu (mmol/L)	5.01 (4.41–6.02)	5.32 (4.85–9.24)	4.80 (4.34–5.64)	0.202
UA (umol/L)	346.3 ± 139.0	354.8 ± 145.0	337.9 ± 136.5	0.721
K (mmol/L)	4.30 (4.10–4.73)	4.36 (4.09–4.76)	4.20 (4.09–4.73)	0.670
Ca (mmol/L)	2.18 ± 0.18	2.17 ± 0.15	2.18 ± 0.21	0.861
P (mmol/L)	1.60 ± 0.55	1.56 ± 0.64	1.64 ± 0.45	0.634
PTH (pmol/L)	30.5 (16.2–39.6)	30.5 (19.3–41.4)	27.8 (14.8–40.0)	0.899
DPI (g/kg/day)	1.07 ± 0.14	1.06 ± 0.13	1.08 ± 0.16	0.645
DEI (kcal/kg/day)	30.25 ± 3.83	29.17 ± 3.22	31.62 ± 4.20	0.062
TSF (mm)	16.0 (15.0–18.0)	16.0 (14.3–20.5)	16.5 (15.7–18.0)	0.542
MAC (cm)	24.6 (22.6–26.0)	25.0 (21.0–27.0)	24.3 (23.7–25.9)	0.891

Note: Data presented as median (first–third interquartile range) or mean ± SD or number (percentage). * represents *p* < 0.05 and ** represents *p* < 0.01. Abbreviations: BMI, body mass index; MQSGA, modified quantitative subjective global assessment; MIS, malnutrition-inflammation score; Hb, hemoglobin; Alb, serum albumin; TC, cholesterol; LDL, low-density lipoprotein; TG, triglycerides; HDL, high-density lipoprotein; Cr, serum creatinine; BUN, blood urea nitrogen; Glu, blood glucose; UA, uric acid; K, serum kalium; Ca, serum calcium; P, serum phosphorus; PTH, parathyroid hormone; DPI, dietary protein intake; DEI, dietary energy intake; TSF, skin-fold thickness; MAC, mid-arm circumference.

### Effect of oral energy supplements on biochemical parameters

**FIGURE 2:**
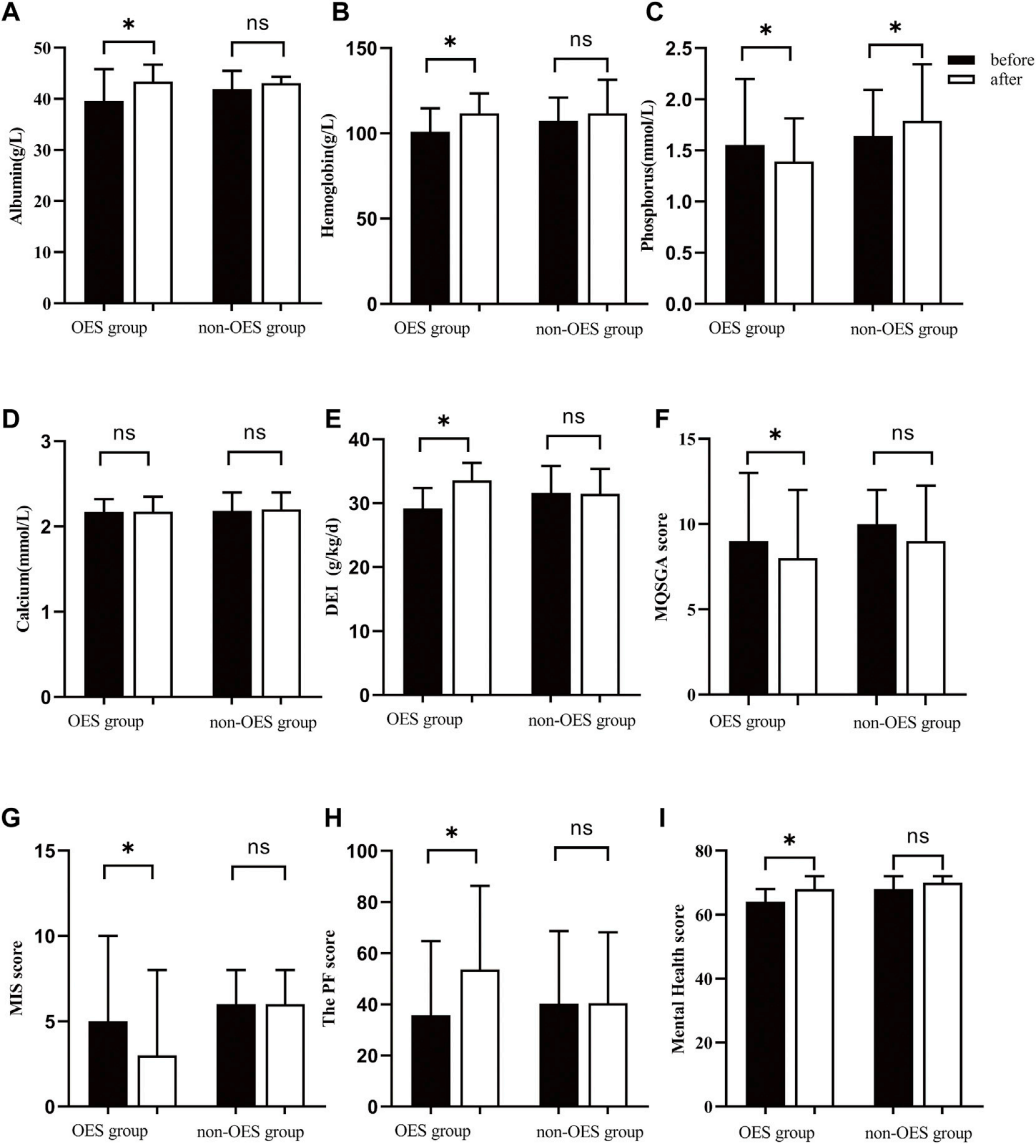
Mean/median changes in total Alb **(A)**, Hb **(B)**, P **(C)**, Ca **(D)**, DEI **(E)**, MQSGA **(F)**, MIS **(G)**, and SF36 **(H,I)** after nutritional intervention. * represents *p* < 0.05.

It could be observed that the Alb levels were increased in both groups during the treatment period. However, a significant difference was only noticed in the OES group [39.6 (37.6–45.8) vs. 43.4 (39.1–46.7) g/L, *p =* 0.036], while not in the non-OES group [41.9 (36.2–45.5) vs. 43.1 (38.4–43.1) g/L, *p =* 0.629] (Figure 2A). Surprisingly, patients treated with OES showed, compared to baseline, an increase in Hb (101.0 ± 13.6 vs. 111.8 ± 11.7 g/L; *p* < 0.001) (Figure 2B). Moreover, a significant decrease in serum P in the OES group (1.56 ± 0.64 vs. 1.39 ± 0.42 mmol/L; *p =* 0.039) was observed, indicating that OES can improve the nutritional status of patients without causing hyperphosphatemia. On the contrary, a statistically significant increase in serum P (1.64 ± 0.45 vs. 1.79 ± 0.55 mmol/L; *p =* 0.01) and a non-statistically significant increase in serum Ca (2.18 ± 0.21 vs. 2.20 ± 0.20 mmol/L; *p =* 0.57) were observed in the non-OES group (Figures 2C,D). This indicates that patients should consume more protein to reverse PEW; however, this will lead to hyperphosphatemia and worse chronic kidney disease-mineral and bone disorder (CKD-MBD) status, suggesting that simply increasing protein intake to prevent PEW may not be suitable for HD patients. No differences in HDL, LDL, TG, TC, UA, Glu, and PTH were observed between the two groups at the baseline compared with those at 2 months (Table 2), which indicated that although the major component of OES we used was fat, it did not affect the lipid profile of patients.

**TABLE 2:** Change in biochemical parameters, food intake, and body composition between the two groups.

Variable	OES group		Non-OES group	
	Before	After	Before	After
Alb (g/L)	39.6 (37.6–45.8)	43.4 (39.1–46.7)*	41.9 (36.2–45.5)	43.1 (38.4–43.1)
Hb (g/L)	101.0 ± 13.6	111.8 ± 11.7**	107.4 ± 13.6	111.9 ± 19.6
TG (mmol/L)	1.14 (0.74–1.80)	1.37 (1.11–1.75)	1.63 (1.03–2.01)	1.26 (0.97–1.75)
HDL (mmol/L)	0.97 (0.78–1.33)	1.09 (0.89–1.39)	0.96 (0.79–1.25)	1.04 (0.92–1.33)
LDL (mmol/L)	1.75 ± 0.69	1.68 ± 0.69	2.02 ± 0.64	1.86 ± 1.02
TC (mmol/L)	3.35 ± 0.678	3.44 ± 0.65	3.87 ± 0.92	3.42 ± 0.61
Cr (umol/L)	679.1 ± 262.6	660.9 ± 151.0	638.0 ± 236.6	844.8 ± 237.2
BUN(mmol/L)	20.5 ± 9.0	17.7 ± 5.9	17.4 ± 8.3	20.8 ± 5.8
Glu (mmol/L)	5.32 (4.85–9.24)	5.42 (4.48–8.19)	4.80 (4.34–5.64)	4.63 (4.30–5.20)
UA (mmol/L)	354.8 ± 145.0	358.8 ± 108.2	337.9 ± 136.5	410.1 ± 127.8
K (mmol/L)	4.36 (4.09–4.76)	4.50 (3.85–4.84)	4.20 (4.09–4.73)	5.00 (4.54–5.76)
Ca (mmol/L)	2.17 ± 0.15	2.17 ± 0.17	2.18 ± 0.21	2.20 ± 0.20
P (mmol/L)	1.56 ± 0.64	1.39 ± 0.42*	1.64 ± 0.45	1.79 ± 0.55*
PTH (pmol/L)	30.5 (19.3–41.4)	30.5 (19.3–41.4)	27.8 (14.8–40.0)	21.2 (19.1–28.9)
DPI (g/kg/d)	1.06 ± 0.13	1.08 ± 0.12	1.08 ± 0.16	1.09 ± 0.13
DEI (kcal/kg/d)	29.17 ± 3.22	33.60 ± 2.72**	31.62 ± 4.20	31.4 ± 3.9
BMI (kg/m^2^)	23.0 (20.9–26.2)	23.0 (21.0–25.5)	22.5 (20.5–23.9)	22.0 (21.1–23.8)
TSF (mm)	16.0 (14.3–20.5)	16.0 (14.4–20.4)	16.5 (15.7–18.0)	16.5 (15.7–18.3)
MAC (cm)	25.0 (21.0–27.0)	25.0 (22.0–26.0)	24.3 (23.7–25.9)	24.6 (23.4–25.9)

Note: Data presented as median (first–third interquartile range) or mean ± SD, or number (percentage). * represents *p* < 0.05 and ** represents *p* < 0.01. Abbreviations: Cr, serum creatinine; BUN, blood urea nitrogen; Glu, blood glucose; Hb, hemoglobin; Alb, serum albumin; TG, triglycerides; TC, cholesterol; HDL, high-density lipoprotein; LDL, low-density lipoprotein; UA, uric acid; PTH, parathyroid hormone; K, serum kalium; Ca, serum calcium; P, serum phosphorus; BMI, body mass index; DPI, dietary protein intake; DEI, dietary energy intake; TSF, skin-fold thickness; MAC, mid-arm circumference.

### Effect of oral energy supplements on protein, energy, body composition, and nutrition score

It was found that the DEI was apparently increased in the OES group (29.17 ± 3.22 vs. 33.60 ± 2.72 kcal/kg/day, *p* < 0.001), while not in the non-OES group (31.62 ± 4.20 kcal/kg/day vs. 31.4 ± 3.9 kcal/kg/day, *p =* 0.086) (Figure 2E). Surprisingly, we noticed that the DEI was decreased a little in the non-OES group after dietary recommendations. After consulting with the clinical dietitian, this was found to be caused by the insufficient fat intake in HD patients. Based on these results, we believed that in HD patients with PEW, just dietary recommendations may not be enough to improve the energy intake, but the addition of OES was necessary. Further analysis suggested that no significant differences were found in the DPI in both the groups before and after treatment. No differences in BMI, MAC, and TSF were observed between the two groups (Table 2). This may be due to the short duration of treatment. Moreover, remarkable improvements in MQSGA [9 (8–13) vs. 8 (7–12), *p* < 0.001] and MIS were documented [5 (3–10) vs. 3 (2–8), *p* < 0.001], whereas no significant changes were documented at the baseline compared with those at 2 months in the non-OES group (Figure 2F&G, and Table 3).

**TABLE 3:** Change in SF36, MQSGA, and MIS between the two groups.

	OES group		Non-OES group	
	Before	After	Before	After
SF36				
PF	35.8 ± 29.0	53.7 ± 32.7 **	40.4 ± 28.3	40.6 ± 27.7
RF	0 (0–0)	0 (0–0)	0 (0–0)	0 (0–0)
BP	68.2 ± 24.7	72.3 ± 22.7	68.9 ± 18.6	71.7 ± 21.6
GH	34.1 ± 9.3	31.7 ± 9.1	34.1 ± 7.6	32.8 ± 9.2
V	61.8 ± 8.4	59.7 ± 10.1	62.5 ± 9.6	61.1 ± 12.4
SF	75 (50–88)	88 (63–88)	81 (50–88)	75 (59–88)
RE	0 (0–0)	0 (0–0)	0 (0–0)	0 (0–0)
MH	64 (64–68)	68 (64–72) *	68 (63–72)	70 (63–72)
MIS	5 (3–10)	3 (2–8) **	6 (2–8)	7 (3–8)
MQSGA	9 (8–13)	8 (7–12) **	10 (8–12)	9 (8–12)

Note: Data presented as median (first–third interquartile range) or mean ± SD, or number (percentage). * represents *p* < 0.05 and ** represents *p* < 0.01. Abbreviations: SF-36, Short Form Health Survey Questionnaire; MQSGA, modified quantitative subjective global assessment; MIS, malnutrition-inflammation score; PF, physical functioning; RF, role-physical; BP, bodily pain; GH, general health; V, vitality; SF, social functioning; RE, role-emotional; MH, mental health.

### Effect of oral energy supplements on SF36

Previous studies have reported that malnutrition can adversely affect the quality of life (Nagy et al., 2021). In our study, a significant amelioration was observed in physical functioning (35.8 ± 29.0 vs. 53.7 ± 32.7; *p* < 0.001) and mental health [64 (64–68) vs. 68 (64–72); *p =* 0.046] subsections of the (SF)-36 health survey in the OES group, whereas no significant changes were observed in the non-OES group (Figures 2H,I and Table 3).

## Adverse events

The most frequent adverse events in the OES group were digestive symptoms, mainly diarrhea, nausea, and abdominal distention, as observed in two patients. In addition, one patient in the OES group died of an acute cerebrovascular event.

## Discussion

PEW, with a prevalence of 18%–75% among the dialysis population, is closely associated with both increased morbidity/mortality risk and worsened quality of life (Sabatino et al., 2017; Carrero et al., 2018). Inadequate caloric intake is a key cause of PEW. Although the KDOQI guidelines recommend that HD patients should consume 30–35 kcal/kg/day of energy (Ikizler et al., 2020), studies have shown that minimum calorie requirements are often not met in patients with CKD, despite extensive nutritional counseling (Wu et al., 2013). Thus, additional interventions are needed to help patients achieve therapeutic nutritional energy goals, whereas the effectiveness of OES in the HD population is lacking.

In the current study, we found that OES could improve the nutrition status in HD patients with PEW. After 2 months of OES treatment, a significant amelioration of the nutrition status (Alb, Hb, DEI, MQSGA, and MIS) and quality of life was observed in the patients in the OES group, who received an additional 300 kcal energy supply daily. Our finding is consistent with that of a recent study using oral nutritional supplements with protein and fat (Limwannata et al., 2021). We also noticed that the serum P level in OES-treated patients decreased significantly after treatment and increased in patients of the non-OES group. Regarding the fact that the ingredients of OES do not contain phosphorus, potassium, and calcium, it is very safe for patients with advanced CKD, especially ESRD. Addition of OES will ameliorate malnutrition in HD patients without worsening CKD-MBD.

Although the OES we used in this study could only supply energy, and not protein, yet the serum albumin level increased apparently after OES supply. This indicates that sufficient energy supply will avoid protein intake burned as calories by replenishing energy, thus achieving a positive nitrogen balance and ameliorating hypoalbuminemia.

Therefore, we believe that this kind of OES is also suitable for patients who need a low-protein diet, especially CKD four or pre-dialysis CKD five patients. In this study, OES did not significantly change the BMI, TSF, and MAC in HD patients after 2 months of treatment, which was similar to the observation of the previous study (Li et al., 2020). It may be due to the limited observation time. We do believe that prolonged treatment will benefit the body composition of patients.

Dyslipidemia, which is correlated to cardiovascular events, is common in patients with chronic kidney disease (Wang et al., 2020). However, hemodialysis patients often aggravate dyslipidemia by increasing dietary intake. According to the literature review, unsaturated fatty acids have the tendency to reduce the burden of CVDs (Jayachandran et al., 2020). OES used in this study contains a high proportion of fat, but all are unsaturated fatty acids; thus, no lipid metabolism disorders are found in our study.

Anemia occurs in more than 90% of the ESRD patients who undergo dialysis. Many factors contribute to renal anemia, including a combination of erythropoiesis-stimulating agents (ESAs) hyporesponse and resistance (Weir, 2021), chronic inflammation, uremic toxins, disturbed iron homeostasis, and malnutrition (Borawski et al., 2021). In our study, the average concentration of Hb after supplementation was increased compared with that of the baseline. Improvement in nutritional status can increase the response for ESAs and thus ameliorate anemia. Thus, in the future, recognizing energy supplements may be a potential treatment strategy for patients ameliorating anemia, and patients who are mildly malnourished but with severe anemia may benefit from this energy supplement. Further research is needed to verify this.

Emerging evidence has shown that malnutrition is associated with poor quality of life, complications, and an increased risk of mortality (Qureshi et al., 2002). However, whether OES can improve the quality of life remains unknown. The results of our cohort study showed a significant increase in scores in physical functioning and mental health in comparison with the baseline, thereby confirming that OES can improve the quality of life of ESRD patients on chronic hemodialysis. According to the literature review, lack of energy is associated with impaired physical function (Martin et al., 2011). The amelioration of energy supply, nutritional status, and anemia could improve physical endurance, thus improving physical function. However, no significant differences were observed in other dimensions, which might be due to the limited observation time. Long-term follow-up is required to identify our results in the future.

The limitations of this study should be recognized. First, the average follow-up time in our study was relatively short. Second, it is recommended to develop clinical management protocols for patients with chronic kidney disease, which includes the assessment of physical function or encouragement of physical activity (Aucella et al., 2014). However, the levels of physical activity were not assessed and lacked some important experimental parameters, such as prealbumin and C-reactive protein, in the current study. Moreover, no prespecified hypothesis in regard to the statistical significance and the correlations was assumed, and no sample size to obtain a given power was calculated. It is a political study for exploration. Thus, the sample size is limited. The results might not necessarily be representative of those of other countries and regions. Nevertheless, this is one of the few studies that explore the relationship between OES and the quality of nutritional status of HD patients, providing insight into nutritional intervention as studied under routine clinical practice conditions. In the near future, we will expand the sample size, and we are planning to conduct further multicentric, prospective randomized controlled trials to identify our results.

## Conclusion

Our study has demonstrated that OES intake in patients undergoing hemodialysis can significantly ameliorate energy supply, nutritional status, anemia, and quality of life.

## Data Availability

The raw data supporting the conclusion of this article will be made available by the authors, without undue reservation.
